# Nutritional Composition and Untargeted Metabolomics Reveal the Potential of *Tetradesmus obliquus*, *Chlorella vulgaris* and *Nannochloropsis oceanica* as Valuable Nutrient Sources for Dogs

**DOI:** 10.3390/ani12192643

**Published:** 2022-10-01

**Authors:** Ana R. J. Cabrita, Joana Guilherme-Fernandes, Inês M. Valente, Agostinho Almeida, Sofia A. C. Lima, António J. M. Fonseca, Margarida R. G. Maia

**Affiliations:** 1 REQUIMTE, LAQV, ICBAS, Instituto de Ciências Biomédicas Abel Salazar, Universidade do Porto, Rua de Jorge Viterbo Ferreira 228, 4050-313 Porto, Portugal; 2 REQUIMTE, LAQV, Departamento de Química e Bioquímica, Faculdade de Ciências, Universidade do Porto, Rua do Campo Alegre 687, 4169-007 Porto, Portugal; 3 REQUIMTE, LAQV, Departamento de Ciências Químicas, Laboratório de Química Aplicada, Faculdade de Farmácia, Universidade do Porto, Rua de Jorge Viterbo Ferreira 228, 4050-313 Porto, Portugal

**Keywords:** microalgae, nutritional composition, metabolomic, dog, sustainability

## Abstract

**Simple Summary:**

The growing pet population is fuelling the debate over the environmental impact of the pet food sector and questioning its sustainability, unveiling the need for alternative and more sustainable ingredients. The present study relates the nutritional composition and metabolomic profile of the microalgae *Tetradesmus obliquus*, *Chlorella vulgaris*, and *Nannochloropsis oceanica* to the nutritional requirements of dogs to assess their potential as alternative foods. Overall, the essential amino acid content exceeded the amounts required for dogs at all life stages, except methionine and cysteine, and their different fatty acid and mineral profiles demonstrates a different potential for dog feeding. In addition, fiber was mainly composed of insoluble dietary fiber and untargeted metabolomics highlighted glycolipids, glycerolipids and phospholipids as the most discriminating compounds. The results support the potential of *T. obliquus*, *C. vulgaris* and *N. oceanica* as valuable nutrient sources for dogs.

**Abstract:**

The growing pet population is questioning the sustainability of the pet food system. Although microalgae may constitute a more sustainable food resource, the assessment of their potential for canine diets is almost non-existent. The present study aimed to evaluate the potential of three microalgae species (*Tetradesmus obliquus*, *Chlorella vulgaris* and *Nannochloropsis oceanica*) grown locally in industrial photobioreactors as alternative food resources for dogs. A detailed characterization of their nutritional composition and metabolomic profile was carried out and related to the nutritional requirements of dogs. Overall, the essential amino acid content exceeded the amounts required for dogs at all life stages, except methionine and cysteine. The three microalgae were deficient in linoleic acid, *N. oceanica* presented a linolenic acid content below requirements and *T. obliquus* and *C. vulgaris* were deficient in arachidonic and eicosapentaenoic acids. The fiber was mainly composed of insoluble dietary fiber. The mineral profile varied greatly with the microalgae species, demonstrating their different potential for dog feeding. Untargeted metabolomics highlighted glycolipids, glycerolipids and phospholipids as the most discriminating compounds between microalgae species. Overall, the results support the potential of *T. obliquus*, *C. vulgaris* and *N. oceanica* as valuable macro- and micro-nutrients sources for dog feeding.

## 1. Introduction

After a long history of domestication, dogs are now considered family members with recognized beneficial effects on human physical and psychological health [1]. By the year 2020, 70% of households in the USA reported having at least one pet, while in Europe 38% of all households had at least one pet, reaching a total of 89.7 [2] and 89.8 [3] million dogs, respectively. The global pet population is expected to keep pace with the growth of the human population, which, at the current rate, will reach 9.8 billion by 2050. This is fueling debate over the environmental impact of the pet food sector and questioning its sustainability. In fact, the pet food industry comprises a long chain of activities responsible for energy and natural resources use, waste production and greenhouse gas emission [4], and competes with humans and livestock for food resources [5]. Additionally, the increased humanization and anthropomorphism of dogs has led to a growing demand for these animals’ welfare and health with owners pursuing optimal nutrition that meets energy and nutrient requirements, and has functional effects [6]. To keep up with these trends, more sustainable and functional food sources are needed.

Microalgae may constitute an underexploited food resource to achieve these goals. Microalgae are unicellular photosynthetic microorganisms capable of converting inorganic and organic carbon sources into nutrient-rich biomass, in a process up to 10 times more efficient than that of terrestrial plants, and requiring less land and water resources [7]. There is a wide variety of species with a great diversity of characteristics due to the multiplicity of habitats and growing conditions, such as temperature, light, pH and nutrient availability [8]. Regardless of the species and environmental conditions, in general, microalgae are rich in macro- and micronutrients, and in bioactive compounds such as proteins and peptides, lipids, namely polyunsaturated fatty acids, and sterols, pigments and polysaccharides [9]. To harness the potential of microalgae as ingredients in pet food, their large-scale and local production is crucial to ensure market availability at reasonable prices and the sustainability of the food sector [10]. In recent decades, the large-scale cultivation of some microalgae has expanded, with about 19% being used as animal feed in Europe [10]. In terms of the number of companies, *Chlorella* sp., *Nannochloropsis* sp. and *Tetradesmus* sp. are among the top produced species in Europe [10], with *Chlorella* sp. and spirulina (*Arthrospira* sp.) the most produced species in terms of dry algae mass [11]. Microalgae have been studied mainly as components of aquafeeds, with their use in pet food being scarcely evaluated. Thus, the aim of the present study was to assess the potential of three microalgae species (*Tetradesmus obliquus*, *Chlorella vulgaris* and *Nannochloropsis oceanica*), produced locally in an industrial setting, as valuable nutrient resources for complete and balanced pet food through a detailed characterization of their nutritional composition and untargeted metabolomic profile.

## 2. Materials and Methods

### 2.1. Microalgae Species

Three commercially available microalgae species (*T. obliquus*, *C. vulgaris* and *N. oceanica*) were studied. All species were produced locally in photobioreactors and kindly provided by Allmicroalgae-Natural Products, S.A. (Pataias, Portugal) as a spray dried powder in airtight bags protected from sunlight.

### 2.2. Nutritional Composition of Microalgae Species

#### 2.2.1. Proximate Analysis

Proximate composition of the microalgae species was analyzed in duplicate according to official methods [12]. Samples were analyzed for dry matter (DM; ID 934.01), ash (ID 942.05), Kjeldahl N (ID 954.01), crude fiber (CF; ID 978.10) and insoluble and soluble dietary fiber (ID 2011.25). Crude protein (CP) was calculated as Kjeldahl N × 4.78 [13]. Neutral detergent fiber (NDF; with α-amylase and without sodium sulphite) and acid detergent fiber (ADF) were analyzed and expressed excluding residual ash [14]. For fiber analysis, a change in the filtration step was performed due to the small size of the microalgae powder particles, and the hydrolyzed samples were filtered through a glass microfiber filter (Whatman GF/A, 1.6 μm porosity, Merck KGaA, Darmstadt, Germany). Total lipids, starch, and gross energy were determined as earlier described [15].

#### 2.2.2. Amino Acids

Hydrolyzed microalgae samples (6 M HCl solution at 116 °C for 48 h) were precolumn derivatized with Waters AccQ Fluor Reagent (6-aminoquinolyl-N-hydroxysuccinimidyl carbamate) using the AccQ Tag method (Waters, Milford, MA, USA). Analyses were performed by ultra-high-performance liquid chromatography on a Waters reversed-phase amino acid analysis system. Norvaline was used as internal standard. The resulting peaks were analyzed with the EMPOWER software (Waters) [16]. Analyses were performed in duplicate. Amino acid scores (AAS) were calculated [17], using the minimum levels recommended by FEDIAF [18] for complete food for adult dogs (maintenance energy requirement of 110 kcal/kg^0.75^), dogs in early growth (<14 weeks) and reproduction, and late growth (≥14 weeks). The geometric mean of AAS was calculated to determine the index of essential amino acids [19].

#### 2.2.3. Fatty Acids

Microalgal fatty acid methyl esters were prepared by acid-catalyzed transesterification with methanolic HCl [20] and analyzed by gas chromatography as reported by Maia, et al. [21], using a Shimadzu GC-2010 Plus (Shimadzu Corporation, Kyoto, Japan) equipped with a capillary column (Omegawax 250, 30 m × 0.25 mm × 0.25 μm; Supelco, Bellefonte, PA, USA), and a flame-ionization detector. Fatty acids were identified by comparing retention times to standards (Supelco 37 Component FAME Mix, BAME Mix, PUFA No.1, PUFA No.2, PUFA No.3, Sigma-Aldrich, St. Louis, MO, USA; GLC-110 Mixture, Matreya, Pleasant Gap, PA, USA), and quantified with the internal standard (C19:0, nonadecanoic acid; Matreya). Analyses were carried out in duplicate.

#### 2.2.4. Minerals

Minerals and trace elements were determined as previously described [22]. Briefly, microalgae samples were mineralized in a Milestone (Sorisole, Italy) MLS 1200 Mega high-performance microwave digestion unit. Sample solutions were analyzed by inductively coupled plasma-mass spectrometry (ICP-MS) and flame atomic absorption spectrometry (FAAS) using a Thermo Fisher Scientific (Waltham, MA, USA) iCAP Q ICP-MS instrument and a PerkinElmer (Shelton, CT, USA) AAnalyst 200 FAAS instrument, respectively. For FAAS, calibration standards were prepared from 1000 mg L^−1^ single-element standard stock solutions (Fluka, Buchs, Switzerland) by appropriate dilution with HNO_3_ 0.2% (*v*/*v*). For ICP-MS determinations, internal standards and tuning solutions were prepared by appropriate dilution of the following commercial solutions: Periodic table mix 3 for ICP-MS (TraceCERT^®^, Sigma-Aldrich, Buchs, Switzerland) containing 10 mg L^−1^ of 16 elements (Sc, Y, la, Ce, Pr, Nd, Sm, Eu, Gd, Tb, Dy, Ho, Er, Tm, Yb and Lu in 5% HNO_3_) and a custom solution (SCP Science, Baie D’Urfé, QC, Canada) with 1 mg L^−1^ of Ba, Bi, Ce, Co, In, Li and U in 5% HNO_3_ + 0.5% HCl, respectively. Analyses were performed in triplicate.

### 2.3. Untargeted Metabolomic Profiling

#### 2.3.1. Microalgae Extraction

Microalgae extracts were prepared as reported by Monteiro, et al. [23]. Briefly, 1 g of dried samples (1 h at 65 °C) were suspended in 20 mL of methanol 80% (*v*/*v*), incubated in a sonication bath for 30 min, in the dark, and filtered with a regenerated cellulose syringe filter (0.45 μm porosity, Tecnocroma, Portugal). The supernatant was collected and the pellets re-extracted twice. The extractions were performed in triplicate.

#### 2.3.2. HPLC-MS/MS

The characterization of the extracts was performed in a high-resolution mass spectrometer coupled to a liquid chromatography system (HPLC-MS/MS) (Vanquish Core, Thermo Scientific, Waltham, MA, USA) with HESI source (Orbitrap Exploris 120 Mass Spectrometer, Thermo Scientific). Chromatographic separation was performed as described by Valente et al. [24]. The HESI source was used in negative ion mode with a capillary temperature of 300 °C, a spray voltage of 2.5 kV, the sheath gas (N_2_) flow at 50 arbitrary units and the auxiliary gas (N_2_) flow at 10 arbitrary units. Mass detection was performed in the range 100–1000 m/z. Data acquisition was performed using the Thermo Scientific Chromeleon Chromatography Data System (Thermo Electron Corporation, Waltham, MA, USA).

#### 2.3.3. Processing of Raw HPLC-MS/MS

The raw files obtained from the HPLC-MS/MS analyses were processed using MZmine 2.53 [25], using the parameters presented in the Supplementary Information (Section S1). The resulting peak list was exported as a csv file for statistical analysis. The identification of compounds was performed by comparison with information from the literature.

### 2.4. Statistical Analysis

For metabolomic data, the peak list generated by MZmine and containing the identified metabolites was uploaded to MetaboAnalyst 5.0 [26] for statistical analysis (ANOVA one factor analysis). Features with missing values were replaced by the detection limits (1/5 of the minimum positive value of each variable). Data were filtered considering the interquartile range to eliminate variables that are near-constant values throughout the experiment. Data were transformed by square root and scaled by Pareto scaling. The heatmap showing the distribution of metabolites in the three microalgae species was constructed using the top 25 metabolites resulting from the Partial Least Squares Discriminant Analysis, based on the variable importance in the projection score.

## 3. Results and Discussion

The studied microalgae species were selected from their local production in an industrial setting and their potential interest as an alternative and sustainable food for dogs. The freshwater mixotrophic *Chlorella* sp. is one of the most cultivated microalgae and the first species produced for food [27]. *Nannochloropsis oceanica* is a fast-growing marine microalga known to accumulate lipids intracellularly [28], being an interesting source of eicosapentaenoic acid (EPA, 20:5 *n*-3), a polyunsaturated fatty acid (PUFA) with known benefits to human and animal health [29]. The green microalgae *T. obliquus* is one of the most studied species due to its rapid growth rate and ability to adapt to stressful conditions and use of various carbon sources [30]. The proximate composition of the studied microalgae is shown in Table 1.

### 3.1. Protein and Amino Acid Profile

Animal proteins and animal by-products are the protein sources traditionally used in pet food. However, the increase in meat consumption due to the growth of human population imposes the search for alternative sources of protein for animal feeding in order to increase the sustainability of the food system. Depending on the life stage, the CP (N × 6.25) requirement levels for complete food for dogs range from 180 to 250 g kg^−1^ DM [18]. Crude protein content was higher in *C. vulgaris* (439 g kg^−1^ DM) and *T. obliquus* (411 g kg^−1^ DM) than in *N. oceanica* (246 g kg^−1^ DM) (Table 1), similar to the values reported in previous studies [9,31]. Thus, a dietary inclusion level of 46.5%, 43.6%, and 77.6%, respectively, of *T. obliquus*, *C. vulgaris* and *N. oceanica*, ensures the highest CP requirements (early growth, <14 weeks of age, and reproduction). Conventionally, the CP content is calculated using the conversion factor 6.25, which in the case of microalgae can lead to an overestimation of the true protein content due to the presence of non-protein nitrogen such as pigments, nucleic acids and other inorganic components [32]. In the present study, CP was calculated using the average factor of 4.78 estimated by Lourenço et al. [13] for 12 different strains of algae grown under a range of environmental conditions. However, it is known that the conversion factor varies with the microalgae strain and growth conditions, such as the N-content of the medium [13] and the characteristics of the cell wall, as it can prevent the solubilization of intracellular proteins [32]. As it is practically impossible to establish conversion factors for all species and growth conditions, 4.78 remains the most recommended value for calculating total protein content from N content, but care must be taken when comparing data from different studies and between total protein and amino acids content. Indeed, CP content of *C. vulgaris* and *N. oceanica* calculated using the 4.78 conversion factor was lower than the analyzed total amino acids content (439 and 246 vs. 510 and 262g kg^−1^ DM, respectively; Table 1 and Table 2), highlighting the importance of further studies to establish adequate conversion factors for different microalgae species and growth conditions. The total amino acid content in *T. obliquus* (358 g kg^−1^ DM) was intermediate among the studied species (Table 2). Among the essential amino acids, arginine, lysine and leucine were present in higher concentrations in all microalgae and histidine in the lowest concentration. Aspartic acid, glutamic acid, alanine and glycine were the non-essential amino acids present in higher concentrations in all microalgae species studied, and cystine was the amino acid found in the lowest amounts (Table 2). The amino acid content and profile of microalgae species is reported to be affected by growth conditions, including carbon source and level, N availability, and mineral levels [33].

Protein sources traditionally used in pet foods, such as meat (e.g., beef, pork, chicken) and fish (e.g., salmon) have a higher content of essential amino acids than microalgae [34]. The high lysine content of the microalgae species studied complements the amino acid profile of cereal grains, generally with a lower content of lysine and higher content of methionine [35]. Taking as a reference the minimum recommended levels of amino acids in complete foods for dogs at all life stages [18], AAS were greater than 100, exceeding the required amounts, with the exception of methionine and the sum of methionine and cystine in all microalgae, the sum of phenylalanine and tyrosine in *T. obliquus* and *N. oceanica*, and histidine in *T. obliquus* (Table 3), thus requiring supplementation with an additional protein source when used in canine diets.

Methionine, an essential sulfur-containing amino acid for dogs, is often the first or second limiting amino acid for dogs fed diets with soybean or rendered meat meals [36]. This amino acid is a constituent of proteins, a methyl donor, an integral part of one-carbon metabolism, and a precursor of cysteine, therefore also of glutathione and taurine. When feeding low sulfur amino acids diets, the rate of taurine synthesis can be compromised, not meeting the requirements of dogs, and might result in dilated cardiomyopathy in the long-term [37]. Phenylalanine is required for the synthesis of protein, tyrosine, and catecholamines involved in the stress response [38]. Being both amino acids (phenylalanine and tyrosine) involved in eumelanin synthesis, when fed deficient diets, black-coated dogs can develop yellowish pigmentation [39]. Dogs fed a histidine-deficient diet had decreased plasma and muscle histidine levels, muscle carnosine, body weight, haematocrit, serum albumin, whole blood zinc and copper concentrations, reduced activity and listlessness [40].

### 3.2. Lipids and Fatty Acid Profile

Lipids in microalgae comprise neutral lipids such as triacylglycerols and free fatty acids, considered as a biofuel source, and polar lipids such as glycolipids and phospholipids, the main components of chloroplasts and membrane lipids, with reported health-promoting effects (e.g., antiviral, antitumor, anti-inflammatory) [41]. Growth conditions greatly affect the lipid content of microalgae, as under environmental stress microalgae cell division ceases and the production of lipids for energy storage is promoted [42]. The total lipid content (Table 1) was higher in *N. oceanica* (140 g kg^−1^ DM), intermediate in *C. vulgaris* (97.9 g kg^−1^ DM) and lowest in *T. obliquus* (83.8 g kg^−1^ DM). Although fat per se is not essential for dogs, as long as the minimum requirement for all essential fatty acids is met, FEDIAF [18] recommends a dietary fat level for complete foods of 55 to 85 g kg^−1^ DM, depending on the life stage. With the exception of *T. obliquus*, the other studied species comply with the recommendations. The fat content of dry dog foods generally ranges from 80 to 180 g kg^−1^ DM, with high fat contributing to increase the palatability of the food but also the risk of developing obesity [43].

The total and individual fatty acid contents of the studied microalgae species are shown in Table 4. The sum of saturated fatty acids (SFA) ranged from 18.2 g kg^−1^ DM in *T. obliquus* to 26.4 g kg^−1^ DM in *N. oceanica*, with palmitic acid (C16:0) being the SFA present in higher concentrations (11.7–18.8 g kg^−1^ DM). Monounsaturated fatty acids (MUFA) were present in concentrations varying between 6.96 and 27.2 g kg^−1^ DM, respectively, in *C. vulgaris* and *N. oceanica*, with oleic acid (C18:1 *n*-9) being the main MUFA in *T. obliquus* and *C. vulgaris* (3.59 and 2.13 g kg^−1^ DM, respectively) and palmitoleic acid (C16:1 *n*-7) in *N. oceanica* (21.5 g kg^−1^ DM). The PUFA content ranged from 25.8 g kg^−1^ DM in *T. obliquus* to 29.8 g kg^−1^ DM in *C. vulgaris*. Among the PUFA, *T. obliquus* stands out for the highest content on linolenic acid (C18:3 *n*-3), *C. vulgaris* for the content on linolenic acid and linoleic acid (C18:2 *n*-6), and *N. oceanica* for its higher content of EPA and arachidonic acid (ARA, C20:4 *n*-6), in agreement with previous results [44].

Compared to the minimum values recommended for complete foods for dogs at all life stages [18], all the studied microalgae species were deficient in linoleic acid, *N. oceanica* presented a linolenic acid content below the requirements and *T. obliquus* and *C. vulgaris* were deficient in ARA and EPA. Docosahexaenoic acid (DHA, C22:6 *n*-3) was detected only in *T. obliquus*, but in negligible amounts (0.195 g kg^−1^ DM). Previous studies on the effects of *n*-3 PUFA supplementation on dog health have been conflicting. Eicosanoids, such as prostaglandins and leukotrienes, derived from ARA, are potent inducers of inflammation, while those derived from EPA suppress the production of proinflammatory cytokines and can modulate the expression of adhesion molecule [45], with feeding optimal amounts of *n*-3 PUFA being reported to benefit several pathological conditions (e.g., atopy, chronic renal insufficiency) [46,47]. Conversely, a high dietary level of *n*-3 fatty acids might make animal tissues more prone to lipid peroxidation [48], thus being important an adequate supplementation with exogenous antioxidants to prevent the increase in free radicals and lipid-oxidative by-products.

In the present study, the *n*-6/*n*-3 ratio ranged from 0.199 (*T. obliquus*) to 0.579 (*C. vulgaris*), reflecting the high content of *n*-3 fatty acids in the studied microalgae. The optimal ratio of *n*-6/*n*-3 in dog food is contradictory, with studies reporting ratios from 1.4:1 to 10:1 [49,50]. However, the *n*-6/*n*-3 ratio cannot be isolated from total PUFA and individual *n*-3 and *n*-6 fatty acids content, as a decrease in *n*-6 fatty acid intake does not produce similar effects as an increase in *n*-3 fatty acid intake [51].

### 3.3. Fibre and Starch Contents

Nutritionally, fiber is defined as the hydrolytically indigestible partially fermentable components of the feed, and chemically these components comprise a mixture of cellulose, hemicelluloses, lignin and soluble dietary fibers. The most common analytical methods to determine fiber content include CF, NDF, ADF and soluble and insoluble fractions of total dietary fiber. Crude fiber ranged from 33.0 g kg^−1^ DM in *N. oceanica* to 66.6 g kg^−1^ DM in *T. obliquus* (Table 1). This fraction underestimates the true fiber content, as it comprises most of the cellulose, but only a part of the hemicellulose and lignin and no ash, and is therefore not considered a good indicator of feed digestibility [52]. However, the declaration of CF content remains mandatory in animal feed.

Neutral detergent fiber and ADF contents were higher in *T. obliquus* (228 and 119 g kg^−1^ DM), intermediate in *C. vulgaris* (164 and 97.8 g kg^−1^ DM) and lowest in *N. oceanica* (153 and 43.4 g kg^−1^ DM), in agreement with previous results [15,53]. However, there is a lack of data on NDF and ADF contents of microalgae species, mainly related to possible interactions between some algae polysaccharides and detergent solutions, giving ADF values higher than NDF values [54], and the filtration step, where the very low contents of NDF reported in some studies may reflect the use of standard crucibles with a porosity (40–100 μm) greater than that of microalgae (<25 μm) [55]. Neutral detergent fiber and ADF are widely used for estimating the nutritional qualities of feed and forage. The NDF fraction includes cellulose, hemicelluloses, lignin, silica and cutins, and ADF includes lignin, cellulose, silica and insoluble forms of nitrogen, but not hemicelluloses. Therefore, these fractions estimate insoluble dietary fiber but not soluble dietary fiber. As soluble and insoluble dietary fibers have different physiological benefits due to their different structure and composition, this fractionation becomes nutritionally important. In the present study, insoluble dietary fiber constituted most of the total dietary fiber in the three studied microalgae species, in agreement with previous studies with extruded *N. oceanica* and *T. obliquus* [56,57]. In dogs, non-fermentable fiber decreases gastric transit time and diet energy density and increases fecal bulk and moisture, aiding in laxation, whereas fermentable and soluble fiber increase digesta viscosity and satiety, decrease gastric emptying, glucose uptake rate, blood cholesterol concentration and benefits the growth of commensal gut bacteria [58,59].

Starch content was higher in *C. vulgaris* (44.1 g kg^−1^ DM), intermediate in *T. obliquus* (10.7 g kg^−1^ DM) and lowest in *N. oceanica* (0.7 g kg^−1^ DM) (Table 1), similar to previous studies [15,60]. Microalgae starch has been considered an affordable approach to bioethanol production, replacing starch-rich terrestrial plants, as a higher starch content can be achieved by manipulating growing conditions, including nutrient starvation, light intensity, light-dark cycle and carbon dioxide concentration, that can regulate the partitioning of carbon in cells to carbohydrates and lipids [61].

Carbohydrates are not essential for dogs. Although they have a metabolic requirement for glucose, it can be synthesized from amino acids or obtained from carbohydrates digestion due to the high activity of metabolic enzymes involved in glycolysis and gluconeogenesis [62]. Among other factors, starch digestibility is affected by granular morphology and structure and by the amylose:amylopectin ratio. Bednar et al. [63] found, in vitro, using a canine model, that small starch granules were better digested than large granules. Starch molecules are composed of amylose, a linear polymer with α-1,4 glycosidic bonds less susceptible to digestion, and amylopectin, a larger polymer containing α-1,4 and α-1,6 glycosidic bonds, more susceptible to digestion [64]. Depending on the ratio of amylose to amylopectin, starch sources are classified as rapidly digestible, slowly digestible, and resistant to digestion. The first two are completely digested in the small intestine at varying rates, thus increasing the postprandial glucose concentration, and hence the glycemic index [65]. Resistant starch is not digested in the small intestine, but is fermented in the colon originating volatile fatty acids that contribute to systemic energy metabolism and inhibit gastric emptying via the ileo-colonic brake, thus affecting meal transit and digestion [66]. Although knowledge of the physicochemical characteristics of starch sources is important, limited information is available on microalga starch.

### 3.4. Ash and Mineral Profile

It is known that the ash content of microalgae depends on the concentration of minerals in the environment, with marine species showing higher contents than freshwater species [67]. Indeed, *N. oceanica*, a marine species, had a higher ash content than the freshwater species *C. vulgaris* and *T. obliquus* (Table 4), within the wide range reported in the literature [9,68]. The total mineral contents followed that of ash with *N. oceanica* showing higher values (75.1 g kg^−1^ DM) than *T. obliquus* (41.2 g kg^−1^ DM) and *C. vulgaris* (41.3 g kg^−1^ DM) (Table 5). The difference between ash and total mineral content was suggested by Liu [69] to be due to the presence of siliceous material in the algae ash. A total of 34 elements were determined, including essential and non-essential, toxic and rare earth elements (Table 5 and Table 6). The mineral profile varied greatly with the microalgae species, with *T. obliquus* showing the highest Ca:P ratio, *C. vulgaris* the highest Ca, P, Mn, Cu and Zn contents, and *N. oceanica* the highest levels of Na, K, Mg and Se. In addition to species-related differences, it is known that the mineral profile of algae depends on the growth conditions, which may explain the slightly different values obtained, compared to previous reports [70,71,72].

Compared to the minimal requirements for complete foods for dogs at all life stages [18], *T. obliquus* presented values below the requirements for Na (for all life stages except adults), Mg (for adults), Ca, Cu (for all life stages except adults) and Se (for all life stages except adults), while *C. vulgaris* was deficient in Na, Mg (for adults), Ca and Se, and *N. oceanica* presented values lower than the requirements for Ca and Zn. On the other hand, *T. obliquus* and *C. vulgaris* presented a P content above the nutritional maximum recommended for adult dogs. Sodium, along with Cl, is involved in maintaining osmotic pressure and extracellular volume, acid-base balance and conductivity in neural tissues. Given an adequate availability of water, healthy dogs can metabolically adapt to wide variations in Na intake via the rennin-angiotensin-aldosterone system [73]. Severe restriction of Na can increase the activation of the renin-angiotensin-aldosterone pathway and increase K excretion, while an increased Na intake promotes water intake, which may be beneficial in decreasing the risk of oxalate uroliths [73]. Of the three species studied, only *N. oceanica* presented a Na content above the safe upper limit set by NRC [36] of 15 g kg^−1^ DM and the content (29 g Na kg^−1^ DM) reported by Zentek and Meyer [74] as causing vomiting in adult dogs and decreasing food palatability.

Magnesium is an intracellular cation with a key role in multiple vital functions, such as DNA and RNA metabolism, protein synthesis, stability of muscle and nerve cell membranes, lymphocyte proliferation, platelet activation and the mineral structure of bones and teeth. Symptoms of Mg deficiency include anorexia, weight loss, hyperextension of carpal joints and posterior ataxia [36]. *Nannochloropsis oceanica* had the highest Mg content, and although excessive Mg intake is not a practical concern, the NRC [36] sets a safe upper limit of dietary Mg content of 1.7 g kg^−1^ DM.

Commercial dry dog food typically contains Ca above requirements [75], while non-supplemented homemade recipes have been reported to be deficient in Ca [76]. Calcium is necessary for healthy bones and teeth, but it also plays an essential role in blood clotting, transmission of nerve impulses and secretory and membranous activities. Its deficiency can result in nutritional secondary hyperparathyroidism and significant skeletal abnormalities. The Ca:P ratio was below the recommendations in the three studied microalgae species due to their high P content. Excessive P intake has been associated with increased risk of urolith formation and the development of chronic kidney disease [77]. However, the effects of excess P are more harmful when it is in the inorganic form, which is absorbed more readily, than in organic forms, which are less absorbed, due to their binding to proteins [78].

*Tetradesmus obliquus* had a very high Fe content (2986 mg kg^−1^ DM), and taking into account data from Albretsen [79] that mild clinical signs (e.g., vomiting, diarrhea and gastrointestinal bleeding) occur with an Fe intake of 20–60 mg per kg body weight, the daily amount of this microalgae in dog food would be set at 67–200 g.

Copper is a component of enzymes that catalyze oxidation reactions and is involved in connective tissue formation, Fe metabolism and hematopoiesis, hair pigmentation, myelin formation and defense mechanisms against oxidative damage [80]. Symptoms of Cu deficiency include loss of hair pigmentation and hyperextensions of the distal phalanges [81]. Selenium and zinc are trace elements with important biological functions (e.g., immune response) [82,83]. The low content of Se and Zn in the studied microalgae species may result in deficiency in dogs. Clinical signs of Se deficiency include reduced serum levels of Se and thyroid hormones [84]. Zinc deficiency is more common in growing dogs fed cereal-based diets with high concentrations of substances that bind Zn. Clinical signs of deficiency include poor growth rate and skin lesions [85].

Regarding non-essential minerals, neither FEDIAF [18] nor NRC [36] give recommendations for minimal and maximum daily intake. Taking into account the maximum tolerable level for poultry and swine proposed by NRC [86], none of the studied microalgae raises concerns regarding animal health due to their mineral content.

**Table 6:** Content of non-essential, toxic and rare earth elements in the studied microalgae species (mg kg^−1^ dry matter). Maximum tolerable levels are reproduced with permission from NRC [86].

	Microalgae			Maximum Tolerable Level Poultry/Swine
	*Tetradesmus obliquus*	*Chlorella vulgaris*	*Nannochloropsis oceanica*	
Non-essential trace elements	245	194	256	
Toxic elements				
As	0.150	0.420	1.38	30
Cd	0.110	0.520	0.130	10.0
Pb	0.100	0.090	0.310	10.0
Al	10.7	64.9	21.3	1000
Ba	14.8	8.86	4.77	100
Be	0.020	0.060	0.010	
Mo	5.08	6.02	1.88	100–150
Ni	1.60	1.74	0.980	250
Sb	0.010	0.010	0.010	
Sn	0.160	0.080	0.280	
Sr	86.7	37.2	116	2000
Tl	ND	0.010	0.020	
V	1.05	2.83	5.65	25(growing birds)/10
B	2.55	3.60	82.0	150
Ti	116	60.5	13.3	
Cr	1.21	2.93	1.86	500/100
Co	3.32	0.320	0.500	25/100
Li	ND	ND	1.50	25.0
Rb	1.89	3.27	4.57	
Zr	0.150	0.550	0.140	
Pd	0.010	0.010	ND	
Cs	ND	0.010	0.020	
Rare earth elements				
Ga	0.020	0.060	0.010	
Nb	0.020	0.010	ND	

### 3.5. Untargeted Metabolomic Profile

**Figure 1:**
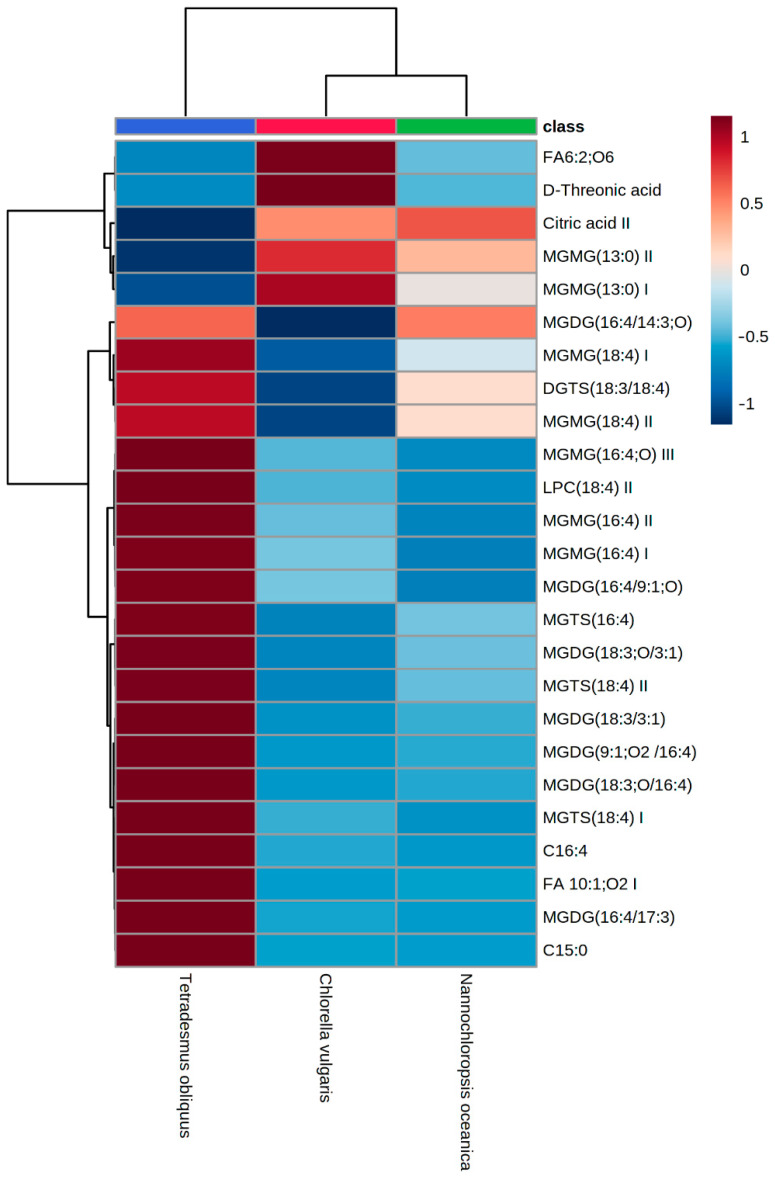
Heatmap showing the distribution of metabolites in the studied microalgae species constructed using the top 25 metabolites resulting from the Partial Least Squares Discriminant Analysis, based on the variable importance in the projection score.

Microalgae are recognized as valuable sources of nutrients and bioactive compounds, most of which not yet identified. To further explore the chemical diversity of the studied microalgae species, an untargeted metabolomic analysis was performed. A total of 220 metabolites were identified in microalgae extracts, distributed by six main chemical classes, including benzoic acids and derivatives, lipids (mono- and diacylglycerols of betaine, glycerophosphocholines, glycerophosphoethanolamines, glycerophosphoinositols, glycosylglycerols, glycosylmono- and diacylglycerols, hydroxy fatty acids, monoacylglycerophosphates, monoacylglycerophosphoethanolamines, and free saturated and unsaturated fatty acids), nucleosides, nucleotides and their analogues (5′-deoxyribonucleosides, purine nucleosides, pyrimidine nucleosides, and ribonucleoside 3′-phosphates), amino acids, peptides and analogues, organic acids, carbohydrates and carbohydrate conjugates (Table S1). The distribution of the top 25 most discriminating compounds among microalgae species is shown in Figure 1 and their identity is described in Table 7.

The metabolic profiles were grouped into two main clusters, with the first cluster composed of *N. oceanica* and *C. vulgaris* and the second of *T. obliquus*. The most discriminating compounds were divided into two regions of interest. From the top, the first region contains (-)-hydroxycitric acid (FA 6:2;O6), *D*-threonic acid, citric acid and monogalactosyl monoacylglycerol (MGMG; 13:0), all present at higher levels in *C. vulgaris*, with citric acid and MGMG (13:0) also present at high levels in *N. oceanica*. In the second region of metabolites, MGMG (18:4, 16:4), monogalactosyl diacylglycerols (MGDG; 16:4/9:1, 18:3/3:1, 9:1/16:4, 18:3/16:4, 18:4/14:3, 16:4/17:3), fatty acids (10:1, 15:0, 16:4), monoacylglycerophosphocholine (LPC; 18:4), diacylglyceryltrimethylhomoserines (DGTS; 18:3/18:4), and monoacylglyceryltrimethylhomoserines (MGTS; 16:4, 18:4) were detected in higher amounts in *T. obliquus*.

Most of the discriminating compounds among microalgae species were polar lipids, namely glycolipids (MGDG), glycerolipids (DGTS) and phospholipids (LPC). Glycolipids are located in the membrane of chloroplasts and thylakoids [87] and their content and composition depend on microalgae species and on growth and environmental conditions [88]. Wang et al. [89] found an increase in MGDG and digalactosyl diacylglycerols in *C. vulgaris* and *T. obliquus* that may indicate an improved membrane structure to counteract osmotic stress. Some glycolipids extracted from microalgae have been reported to have anti-inflammatory activity [90].

Unlike flowering plants, algae have betaine lipids, a family of glycerolipids, in their membranes, with DGTS, diacylglyceryl hydroxymethyl-N,N,N-trimethyl-beta-alanine and diacylglyceryl carboxyhydroxymethylcholine being the main ones [91]. Diacylgyceryl-N-trimethylhomoserine is widely distributed across kingdoms and has been suggested to have similar or even superior phospholipid function in microalgae under phosphate-limited conditions [92]. *N. oceanica* has been shown to remodel lipids by replacing DGTS with LPC under P-limiting stress [93,94]. In this microalgae species, DGTS was found enriched in EPA [91]. In addition, the production of DGTS and MDGD with high EPA content were found to be inversely correlated, suggesting that DGTS and MDGD may comprise two major pools of EPA, partially competing for EPA or its precursors [94]. The DGTS from *N. granulata* have been reported to have anti-inflammatory properties [90].

Lysophosphatidylcholine can present different combinations of fatty acids, with the most common being those with 16, 18 and 20 carbons, with a differentiated impact on their immunomodulatory effects. Pro-inflammatory actions have been attributed to both saturated and monounsaturated LPC, while polyunsaturated forms are reported to be anti-inflammatory [95]. Lysophosphatidylcholines were identified in *N. oceanica* [96], *C. vulgaris* [97] and *T. obliquus* [98].

Threonic acid, a product of ascorbic acid oxidation, was found to accumulate in response to salt stress in *Phaeodactylum tricornutum* [99] and *Thalassiosira pseudonana* [100]. To the best of our knowledge, no effects of threonic acid intake were reported in dogs, but it depressed the ascorbic acid content of testes and liver in guinea pigs [101].

Citric acid, an organic acid involved in tricarboxylic acid cycle, is used as a preservative and to improve the palatability of pet foods [102]. However, the presence of citric acid in petfood was suggested to increase Al absorption in dogs [103].

Hydroxycitric is found in a variety of tropical plants, such as the *Garcinia* species and *Hibiscus sabdariffa* [104,105]. To the best of our knowledge, hydroxycitric acid has never been reported in microalgae. Hydroxycitric acid has been widely used for its anti-obesity effect due to its ability to inhibit ATP-citrate lyase, which is involved in lipogenesis [106]. By preventing lipogenesis, hydroxycitric acid promotes glycogen storage, which may contribute to appetite suppression [107]. Additionally, hydroxycitric acid has been reported to suppress feelings of hunger by increasing serotonin levels [108].

## 4. Conclusions

In conclusion, the present study shows that microalgae produced locally in an industrial setting may provide valuable nutrients in complete and balanced dog foods. In particular, *C. vulgaris* and *T. obliquus* represent protein-rich foods with an amino acid profile that complements amino acids deficiency of cereal-based diets. The three studied microalgae species presented a high content of *n*-3 PUFA and insoluble fiber that can benefit animal health, and their differentiated mineral profile demonstrates their wide potential for dog feeding. In vivo studies are needed to evaluate the biological effects of dietary inclusion of the studied microalgae species and unveil their potential to contribute for a more sustainable dog food system.

## Figures and Tables

**Table 1:** Proximate composition of the spray-dried microalgae species studied (unit per kg of dry matter, DM) ^1^.

	Microalgae		
	*Tetradesmus obliquus*	*Chlorella vulgaris*	*Nannochloropsis oceanica*
DM, g kg^−1^	982	978	988
Ash, g	110	98.9	340
CP, g	411	439	246
Total lipids, g	83.8	97.9	140
NDF, g	228	164	153
ADF, g	119	97.8	43.4
CF, g	66.6	59.8	33.0
IDF, g	229	179	207
SDF, g	7.54	12.5	16.2
TDF, g	237	192	223
Starch, g	10.7	44.1	0.693
GE, MJ	21.5	20.3	16.6

^1^ CP: crude protein; NDF: neutral detergent fiber; ADF: acid detergent fiber; IDF: insoluble dietary fiber; SDF: soluble dietary fiber; TDF: total dietary fiber; GE: gross energy.

**Table 2:** Analyzed content of essential amino acids (EAA) and non-essential amino acids (NEAA) of the studied microalgae species (g kg^−1^ dry matter).

	Microalgae		
	*Tetradesmus obliquus*	*Chlorella vulgaris*	*Nannochloropsis oceanica*
EAA	172	269	130
Arginine	27.2	46.6	22.1
Histidine	4.61	9.51	5.19
Lysine	25.3	53.2	21.9
Threonine	21.7	28.0	14.5
Isoleucine	15.7	21.8	11.3
Leucine	28.4	39.7	20.7
Valine	20.2	29.1	14.8
Methionine	7.67	10.9	5.34
Methionine + cystine	9.16	12.8	6.24
Phenylalanine	21.3	29.9	14.3
Phenylalanine + tyrosine	38.9	57.4	27.2
NEAA	186	242	132
Cystine	1.49	1.94	0.90
Tyrosine	17.6	27.6	12.8
Aspartic acid + Asparagine	31.2	34.9	21.2
Glutamic acid + Glutamine	41.8	52.3	31.3
Alanine	28.6	35.6	17.7
Glycine	27.0	39.2	18.9
Proline	19.1	25.5	15.6
Serine	18.9	24.8	13.3
Total amino acids	358	510	262

**Table 3:** Amino acid scores (AAS) and index of essential amino acids (IEAA) of the studied microalgae species.

	Adults, Based on MER ^1^ of 110 kcal/kg^0.75^			Early Growth (<14 Weeks) and Reproduction			Late Growth (≥14 Weeks)		
	*Tetradesmus obliquus*	*Chlorella vulgaris*	*Nannochloropsis oceanica*	*Tetradesmus obliquus*	*Chlorella vulgaris*	*Nannochloropsis oceanica*	*Tetradesmus obliquus*	*Chlorella vulgaris*	*Nannochloropsis oceanica*
AAS ^2^									
Arginine	175	281	238	154	247	209	137	219	186
Histidine	67.1	130	126	54.9	106	103	68.6	133	129
Lysine	201	397	291	134	263	193	134	265	194
Threonine	140	169	156	124	151	139	126	153	141
Isoleucine	114	149	137	112	146	135	117	152	140
Leucine	116	152	141	102	134	124	132	173	161
Valine	114	155	141	138	187	170	134	181	165
Methionine	64.2	85.3	74.6	102	135	118	110	146	128
Methionine + cystine	33.8	44.9	39.3	50.9	67.7	59.2	53.8	71.5	62.6
Phenylalanine	132	173	148	152	200	171	159	208	178
Phenylalanine + tyrosine	80.2	105	90.0	76.2	100	85.6	79.3	104	89.0
IEAA ^3^	101	145	128	103	147	129	108	155	137

^1^ MER: maintenance energy requirements. ^2^ Calculated according to Kerr, Beloshapka, Morris, Parsons, Burke, Utterback and Swanson [17] using the minimum amino acids levels recommended by FEDIAF [18] for adult dogs (MER of 110 kcal/kg^0.75^), for dogs in early growth (<14 weeks) and in reproduction, and for dogs in late growth (≥14 weeks). ^3^ Calculated according to Oser [19].

**Table 4:** Fatty acid content of the studied microalgae species (g kg^−1^ dry matter) ^1^.

	Microalgae		
	*Tetradesmus obliquus*	*Chlorella vulgaris*	*Nannochloropsis oceanica*
SFA	18.2	19.7	26.4
C8:0	0.006	0.015	0.074
C10:0	0.089	0.073	0.123
C12:0	0.065	0.127	0.340
C14:0	1.51	1.21	4.79
C16:0	11.7	11.8	18.8
C18:0	0.435	1.77	0.343
C20:0	0.040	0.016	0.031
C22:0	0.227	0.096	0.078
C24:0	0.235	0.199	0.050
BCFA	3.39	3.92	1.25
*iso*-C14:0	0.066	0.039	0.030
*iso*-C15:0	0.254	0.671	0.388
*anteiso*-C15:0	0.069	0.163	0.059
*iso*-C16:0	0.045	0.128	0.045
*iso*-C17:0	2.57	2.80	0.716
*anteiso*-C17:0	0.386	0.112	0.016
OCFA	0.536	0.451	0.490
C11:0	0.002	BDL	0.012
C13:0	0.005	0.006	0.016
C15:0	0.221	0.114	0.281
C17:0	0.308	0.330	0.181
MUFA	10.6	6.96	27.2
C14:1 *n*-5	0.019	0.010	0.057
C16:1 *n*-7	1.12	1.21	21.5
C16:1 *n*-9	2.53	2.03	1.02
C17:1 *n*-8	1.47	0.096	0.115
C18:1 *n*-7	1.45	1.36	0.518
C18:1 *n*-9	3.59	2.13	3.82
C20:1 *n*-7	0.015	0.012	0.005
C20:1 *n*-9	0.068	0.035	0.043
C20:1 *n*-11	0.041	0.013	0.018
C22:1 *n*-9	0.033	0.007	0.024
C22:1 *n*-11	0.044	0.045	0.026
C24:1 *n*-9	0.262	0.020	BDL
PUFA	25.8	29.8	26.9
C16:2 *n*-4	0.057	0.088	0.149
C16:3 *n*-4	0.030	0.022	0.174
C16:4 *n*-1	0.050	0.176	0.060
C18:2 *n*-6	3.69	10.4	2.77
C18:2 *n*-6t,t	0.157	0.083	0.038
C18:3 *n*-3	18.6	18.4	0.227
C18:3 *n*-4	0.104	0.051	0.055
C18:3 *n*-6	0.256	0.045	0.263
C18:4 *n*-3	2.01	0.057	0.077
C20:2 *n*-6	0.016	0.055	0.058
C20:3 *n*-3	0.325	0.016	0.091
C20:3 *n*-6	0.015	0.024	0.256
C20:4 *n*-3	BDL	0.020	0.045
C20:4 *n*-6	0.090	0.158	4.60
C20:5 *n*-3	0.169	0.106	17.8
C21:5 *n*-3	0.059	0.079	0.193
C22:2 *n*-6	0.014	0.010	0.017
C22:4 *n*-6	BDL	BDL	BDL
C22:5 *n*-3	BDL	BDL	BDL
C22:6 *n*-3	0.195	BDL	BDL
Total of fatty acids	54.6	56.5	80.4
*n*-6/*n*-3 ratio	0.199	0.578	0.434

^1^ BDL: below the detection limit; SFA: sum of saturated fatty acids; BCFA: sum of branched-chain fatty acids; OCFA: sum of odd-chain fatty acids; MUFA: sum of monounsaturated fatty acids; PUFA: sum of polyunsaturated fatty acids; *n*-6/*n*-3 ratio: ratio of PUFA *n*-6 to PUFA *n*-3.

**Table 5:** Content of essential macro- and trace elements (unit per kg of dry matter) in the studied microalgae species and recommended values for dogs.

	Microalgae			Minimum Recommended Levels for Dogs (Nutritional Maximum) ^1^		
	*Tetradesmus obliquus*	*Chlorella vulgaris*	*Nannochloropsis oceanica*	Adults, Based on MER ^2^ of 110 kcal/kg^0.75^	Early Growth (<14 Weeks) and Reproduction	Late Growth (≥14 Weeks)
Total mineral content, g	41.2	41.3	75.1			
Essential macroelements, g	37.7	39.9	74.4			
Na	1.80	0.500	37.8	1.00	2.20	2.20
K	12.8	8.50	19.1	5.00	4.40	4.40
Mg	0.540	0.560	3.91	0.700	0.400	0.400
Ca	4.36	4.64	0.96	5.00 (25.0)	10.0 (16.0)	8.00–10.0 (18.0)
P	18.2	25.7	12.6	4.00 (16.0)	9.00	7.00
Ca:P ratio	0.239	0.181	0.076	1.00 (2:1)	1.00 (1.6:1)	1.00 (1.6/1.8:1)
Essential trace elements, mg	3213	1190	403			
Fe	2986	644	300	36.00	88.00	88.00
Mn	108	163	35.2	5.80	5.60	5.60
Cu	7.22	25.4	13.6	7.20	11.0	11.0
Zn	113	357	52.5	72.0	100	100
Se	0.310	0.170	1.36	0.180	0.400	0.400

^1^ MER: maintenance energy requirements. ^2^ FEDIAF [18].

**Table 7:** Top 25 metabolites significant for discrimination between microalgae species based on Partial Least Squares Discriminant Analysis.

Metabolite ^1^
MGDG (18:3;O/3:1)
DGTS (18:3/18:4)
(-)-hydroxycitric acid (FA 6:2;O6)
*D*-Threonic acid
MGMG (13:0) II
MGTS (16:4)
FA 10:1;O2 I
MGDG (18:3/3:1)
MGDG (16:4/14:3;O)
MGMG (13:0) I
MGDG (9:1;O2 /16:4)
MGMG (18:4) II
MGDG (18:3;O/16:4)
MGMG (16:4) II
MGTS (18:4) II
MGDG (16:4/17:3)
MGMG (18:4) I
MGMG (16:4;O) III
C15:0
MGTS (18:4) I
LPC (18:4) II
MGMG (16:4) I
Citric acid II
MGDG (16:4/9:1;O)
C16:4

^1^ Roman numerals represent different isomers; FA, fatty acid, LPC, lysophosphatidylcholine, MGMG, Monogalactosyl monoacylglycerol; MGDG, Monogalactosyldiacylglycerol; MGTS, monoacylglyceryl-O-4′-(N,N,N-trimethyl) homoserine; DGTS, diacylglyceryl-O-4′-(N,N,N-trimethyl) homoserine.

## Data Availability

The data presented in this study are available on request from the corresponding author.

## Supplementary Materials

The following supporting information can be downloaded at: https://www.mdpi.com/article/10.3390/ani12192643/s1, Table S1. List of metabolites by class identified in the methanolic extracts of *Tetradesmus obliquus*, *Chlorella vulgaris*, and *Nannochloropsis oceanica* by HPLC-MS/MS, with the information of the ion type, calculated m/z, experimental m/z, mass error (Δm/z) and peak area for each microalgae species. References [109,110] are cited in the supplementary materials.
