# Supplementary material for: Nutritional Composition and Untargeted Metabolomics Reveal the Potential of Tetradesmus obliquus, Chlorella vulgaris and Nannochloropsis oceanica as Valuable Nutrient Sources for Dogs
Source: Animals (Basel). 2022 Oct 1;12(19):2643. doi: 10.3390/ani12192643 (PMC9558554; doi:10.3390/ani12192643)
Supplement: Supplementary file 1 [file animals-12-02643-s001.zip › animals-1895069-supplementary.pdf]

Nutritional composition and untargeted metabolomics reveal the potential of *Tetradesmus obliquus*, *Chlorella vulgaris* and *Nannochloropsis oceanica* as valuable nutrient sources for dogs

Ana R.J. Cabrita [1,*], Joana Guilherme-Fernandes [1], Inês M. Valente [1,2], Agostinho Almeida [3], Sofia A.C. Lima [3], António J.M. Fonseca [1] and Margarida R.G. Maia [1]

Supplementary Information

S1. Processing of raw HPLC-MS data using MZmine

The raw files obtained by HPLC-MS/MS analyses were extracted and converted to mzXML files using the ProteoWizard MSconvert tool [1] and subsequently processed using MZmine 2.53 [2]. Mass detection was performed both in MS^1 level (noise level set at $1x10^5$) and MS^2 level (noise level set at $1x10^4$). The ADAP Chromatogram Builder Module [3] was used for the chromatogram builder using MS^1 (10 scans as minimum group size; group intensity threshold $3x10^5$; minimum highest intensity $3x10^5$; m/z tolerance of 0.01 m/z or 5 ppm). The chromatograms were deconvoluted using the local minimum feature resolver using the following parameters: chromatographic threshold of 30%, minimum search range 0.05 minutes; minimum relative height of 5%; minimum absolute height $1x10^6$; minimum ratio of peak top/edge 5; peak duration range 0.1-2 minutes, m/z range for MS^2 scan pairing (Da): 0.01; RT range for MS^2 scan pairing (min): 0.1. The chromatograms were deisotoped using the isotopic peaks grouper algorithm with a m/z tolerance of 0.01 m/z or 10 ppm and a retention time (RT) tolerance of 0.5 minutes, considering a maximum charge of 2 and the most intense isotope as the representative. Peak alignment was achieved by join aligner using m/z tolerance of 0.01 m/z or 5 ppm (weight for m/z: 75) and RT tolerance of 0.5 minute (weight for retention time: 25). The final peaks list was filtered keeping only the features with MS^2 scan and a minimum of 2 peaks per row. The peak list was gap-filled with the peak finder (multithreaded) module (intensity tolerance at 20%, m/z tolerance of 0.01 m/z or 5 ppm, and absolute RT tolerance of 0.5 min). Fragment peaks were searched considering a RT tolerance of 0.5 minutes, m/z tolerance of MS^2 data of 0.01 m/z or 5 ppm, maximum fragment peak height of 80% and a minimum MS^2 peak height of $1x10^4$. Adducts with formic acid, sodium, sodium formate, chlorine, and complexes were searched using the following parameters: RT tolerance: 0.5; m/z tolerance: 0.01 m/z or 5 ppm; max relative adduct/complex peak height: 80%. Fragments, adducts, and complexes, were removed from the final peaks' list.

Table S1. List of metabolites by class identified in the methanolic extracts of *Tetradesmus obliquus*, *Chlorella vulgaris*, and *Nannochloropsis oceanica* by HPLC-MS/MS, with the information of the ion type, calculated m/z, experimental m/z, mass error (Δm/z), and peak area for each microalgae species.

Metabolite	Ion	Calculated m/z	Experimental m/z	Δm/z (ppm)	*Tetradesmus obliquus*	*Chlorella vulgaris*	*Nannochloropsis oceanica*
				Benzoic acids and derivatives			
Benzoic acid	[M-H]-	121.0295	121.0295	0.0000	8.48E+07	1.24E+08	4.07E+07
				Lipids and lipid-like molecules			
Betaine monoacylglycerols							
MGTS(16:0) I	[M+HCOO]-	518.3698	518.3698	0.0000	2.35E+07	1.02E+06	2.18E+07
MGTS(16:0) II	[M+HCOO]-	518.3698	518.3697	-0.1929	9.59E+07	2.92E+06	8.36E+07
MGTS(16:4)	[M+HCOO]-	510.3072	510.3071	-0.1960	3.00E+07		
MGTS(18:3) I	[M+HCOO]-	540.3542	540.3541	-0.1851	3.08E+07	2.29E+06	
MGTS(18:3) II	[M+HCOO]-	540.3542	540.3541	-0.1851	5.60E+07	3.14E+06	5.68E+06
MGTS(18:4) I	[M+HCOO]-	538.3385	538.3415	5.5727	7.85E+07		
MGTS(18:4) II	[M+HCOO]-	538.3385	538.3413	5.2012	2.18E+08		
MGTS(20:4)	[M+HCOO]-	566.3698	566.3692	-1.0594		7.52E+06	7.01E+07
Betaine diacylglycerols							
DGTS(14:0/16:1)	[M+HCOO]-	726.5526	726.5532	0.8258			4.64E+07
DGTS(18:3/18:4)	[M+HCOO]-	798.5526	798.5521	-0.6261	1.46E+08		1.60E+06
Glycerophosphocholines							
LPC(16:0) I	[M+HCOO]-	540.3307	540.3307	0.0000	1.26E+07	3.00E+07	3.60E+07
LPC(16:0) II	[M+HCOO]-	540.3307	540.3306	-0.1851	5.03E+07	1.30E+08	1.56E+08
LPC(16:2) I	[M+HCOO]-	536.2994	536.2993	-0.1865	1.67E+07	1.83E+08	3.38E+06
LPC(16:2) II	[M+HCOO]-	536.2994	536.2993	-0.1865	5.06E+07	1.54E+08	3.74E+07
LPC(16:3) I	[M+HCOO]-	534.2837	534.2834	-0.5615	3.29E+06	4.40E+08	
LPC(18:1) I	[M+HCOO]-	566.3463	566.3462	-0.1766	2.13E+07	9.19E+07	3.81E+07
LPC(18:1) II	[M+HCOO]-	566.3463	566.3461	-0.3531	9.83E+07	1.31E+08	1.61E+08
LPC(18:2) I	[M+HCOO]-	564.3307	564.3306	-0.1772	4.42E+07	2.70E+08	5.05E+07
LPC(18:2) II	[M+HCOO]-	564.3307	564.3306	-0.1772	1.80E+08	3.68E+08	1.75E+08
LPC(18:3)	[M+HCOO]-	562.3150	562.3149	-0.1778	1.62E+08	5.28E+08	2.98E+06
LPC(18:4) I	[M+HCOO]-	560.2994	560.2993	-0.1642	4.13E+07		
LPC(18:4) II	[M+HCOO]-	560.2994	560.2996	0.3712	1.30E+08		
sn-glycerol-3-phosphocholine	[M+HCOO]-	302.1010	302.1009	-0.3310	5.20E+07	2.47E+08	
Glycerophosphoethanolamines							
PE(16:1/16:1) I	[M-H]-	686.4766	686.4767	0.1355	1.83E+08	1.59E+07	
PE(16:1/16:1) II	[M-H]-	686.4766	686.4766	-0.0102	1.18E+08	2.92E+07	
Glycerylphosphorylethanolamine	[M-H]-	214.0486	214.0486	0.0000	1.38E+08	7.20E+08	
Glycerophosphoinositols							
Glycerophosphoinositol	[M-H]-	333.0592	333.0592	0.0047	1.66E+08	1.47E+09	
Glycosylmonoacylglycerols							
DGMG(14:0) I	[M+HCOO]-	671.3496	671.3499	0.4469	1.06E+07	5.66E+06	5.64E+07
DGMG(14:0) II	[M+HCOO]-	671.3496	671.3486	-1.4895	1.10E+07		6.97E+07
DGMG(16:0) I	[M+HCOO]-	699.3809	699.3807	-0.2860	2.99E+07	7.38E+07	1.00E+08
DGMG(16:0) II	[M+HCOO]-	699.3809	699.3810	0.1430	1.11E+08	3.96E+07	3.70E+08

DGMG(16:3) I	[M+HCOO]-	693.3339	693.3325	-2.0192	4.46E+07	2.44E+08	
DGMG(16:3) II	[M+HCOO]-	693.3339	693.3337	-0.2885	1.66E+08	9.90E+07	1.04E+07
DGMG(18:2) I	[M+HCOO]-	723.3809	723.3808	-0.1382	2.59E+07	3.21E+07	6.46E+06
DGMG(18:2) II	[M+HCOO]-	723.3809	723.3808	-0.1382	4.23E+07	4.33E+07	5.70E+07
MGMG(13:0) I	[M+HCOO]-	495.2811	495.2802	-1.8171		6.43E+07	1.08E+06
MGMG(13:0) II	[M+HCOO]-	495.2811	495.2808	-0.6057		1.10E+08	1.01E+07
MGMG(14:0) I	[M+HCOO]-	509.2967	509.2964	-0.5890	4.50E+06	8.67E+06	1.92E+08
MGMG(14:0) II	[M+HCOO]-	509.2967	509.2964	-0.5890	1.90E+07	8.88E+06	8.41E+08
MGMG(16:0) I	[M+HCOO]-	537.3280	537.3271	-1.6750	8.91E+06	2.73E+07	8.73E+07
MGMG(16:0) II	[M+HCOO]-	537.3280	537.3278	-0.3722	7.64E+07	1.84E+07	2.85E+08
MGMG(16:2) I	[M+HCOO]-	533.2967	533.2964	-0.5625	4.26E+07	7.63E+08	4.94E+06
MGMG(16:2) II	[M+HCOO]-	533.2967	533.2965	-0.3750	1.54E+08	4.58E+08	5.32E+07
MGMG(16:3) I	[M+HCOO]-	531.2811	531.2807	-0.7529	1.56E+08	2.92E+09	2.73E+07
MGMG(16:3) II	[M+HCOO]-	531.2811	531.2809	-0.3764	5.42E+08	1.39E+09	1.36E+08
MGMG(16:3) III	[M+HCOO]-	531.2811	531.2811	0.0000	9.51E+07	9.66E+06	3.25E+07
MGMG(16:4) I	[M+HCOO]-	529.2654	529.2643	-2.0784	1.01E+09	2.30E+06	
MGMG(16:4) II	[M+HCOO]-	529.2654	529.2642	-2.2673	3.86E+09	2.30E+06	
MGMG(16:4;O) I	[M+HCOO]-	545.2603	545.2590	-2.3842	1.02E+08	1.80E+06	
MGMG(16:4;O) II	[M+HCOO]-	545.2603	545.2590	-2.3842	3.16E+08	1.80E+06	
MGMG(16:4;O) III	[M-H]-	499.2548	499.2541	-1.4021	7.95E+07		
MGMG(16:4;O) IV	[M+HCOO]-	545.2603	545.2591	-2.2008	1.41E+08		
MGMG(17:3) I	[M+HCOO]-	545.2967	545.2958	-1.6505	1.46E+07	1.60E+07	
MGMG(17:3) II	[M+HCOO]-	545.2967	545.2966	-0.1834	8.78E+07	2.17E+07	
MGMG(18:3) I	[M+HCOO]-	559.3124	559.3121	-0.5364	1.09E+09	4.38E+08	2.76E+06
MGMG(18:3) II	[M+HCOO]-	559.3124	559.3121	-0.5364	3.09E+09	7.27E+08	5.88E+07
MGMG(18:3;O) I	[M+HCOO]-	575.3073	575.3069	-0.6953	4.84E+07		
MGMG(18:3;O) II	[M+HCOO]-	575.3073	575.3070	-0.5215	2.87E+08	1.22E+07	
MGMG(18:3;O) III	[M+HCOO]-	575.3073	575.3069	-0.6953	1.44E+09	3.76E+07	
MGMG(18:3;O) IV	[M+HCOO]-	575.3073	575.3069	-0.6953	5.44E+07		
MGMG(18:4) I	[M+HCOO]-	557.2967	557.2963	-0.7178	5.65E+07		
MGMG(18:4) II	[M+HCOO]-	557.2967	557.2965	-0.3589	2.81E+08		9.71E+06
MGMG(20:5) I	[M+HCOO]-	583.3124	583.3116	-1.3715	7.80E+06	1.18E+07	3.34E+08
MGMG(20:5) II	[M+HCOO]-	583.3124	583.3120	-0.6857	1.03E+07	1.47E+07	1.65E+09
Glycosylglycerols							
Galactosylglycerol	[M+HCOO]-	299.0984	299.0990	2.0060	5.97E+07	1.99E+08	
Glycosyldiacylglycerols							
DGDG(16:0/4:1)	[M+HCOO]-	767.4071	767.4084	1.6940			5.29E+07
MGDG(10:3;O/20:5)	[M+HCOO]-	747.3961	747.3956	-0.6690			5.99E+07
MGDG(14:0/7:1;O)	[M+HCOO]-	635.3648	635.3670	3.4626			7.71E+07
MGDG(16:1/16:3)	[M+HCOO]-	767.4951	767.4951	0.0000	3.18E+07	2.53E+08	3.12E+07
MGDG(16:1/4:1)	[M+HCOO]-	603.3386	603.3384	-0.3315	2.20E+07	2.83E+06	4.01E+07
MGDG(16:2/16:3) I	[M+HCOO]-	765.4794	765.4792	-0.2613	2.38E+07	5.84E+08	2.51E+08
MGDG(16:3O/18:3)	[M+HCOO]-	807.4900	807.4898	-0.2477	5.85E+07	4.01E+08	6.51E+06
MGDG(16:4/11:1;O)	[M+HCOO]-	711.3961	711.3966	0.7028	7.42E+07	1.00E+06	4.09E+06
MGDG(16:4/14:3;O)	[M+HCOO]-	749.4118	749.4105	-1.7347	6.52E+07		4.51E+07

MGDG(16:4/17:3)	[M+HCOO]-	775.4638	775.4620	-2.3212	4.59E+07		
MGDG(16:4/9:1;O)	[M+HCOO]-	683.3648	683.3650	0.2927	7.39E+07		
MGDG(18:2/16:2)	[M+HCOO]-	795.5264	795.5255	-1.1313	8.21E+07	3.46E+08	8.44E+06
MGDG(18:2/16:3)	[M+HCOO]-	793.5107	793.5092	-1.8903	7.98E+07	4.10E+08	2.49E+08
MGDG(18:2/16:4)	[M+HCOO]-	791.4951	791.4950	-0.1263	1.86E+08	9.39E+08	4.80E+07
MGDG(18:3/16:4)	[M+HCOO]-	789.4794	789.4820	3.2933	9.06E+08	5.09E+06	3.60E+06
MGDG(18:3/16:4;O)	[M+HCOO]-	805.4744	805.4734	-1.2415	9.83E+08	2.21E+07	
MGDG(18:3/16:4;O2) I	[M+HCOO]-	821.4693	821.4684	-1.0956	6.26E+08	2.49E+07	
MGDG(18:3/16:4;O2) II	[M+HCOO]-	821.4693	821.4688	-0.6087	5.93E+07	1.90E+06	
MGDG(18:3/3:1)	[M+HCOO]-	613.3229	613.3227	-0.3261	1.41E+08		
MGDG(18:3/6:0;O4) I	[M+HCOO]-	721.3652	721.3649	-0.4159	1.39E+08	5.08E+07	1.16E+06
MGDG(18:3/6:0;O4) II	[M+HCOO]-	721.3652	721.3656	0.5545	5.06E+08	6.79E+07	1.46E+07
MGDG(18:3/9:2;O)	[M+HCOO]-	711.3961	711.3962	0.1406	6.51E+07	1.74E+06	1.24E+07
MGDG(18:3;O/16:3)	[M+HCOO]-	807.4900	807.4897	-0.3715	4.47E+07	4.20E+08	4.50E+06
MGDG(18:3;O/16:4)	[M+HCOO]-	805.4744	805.4738	-0.7449	1.07E+09		
MGDG(18:3;O/16:4;O)	[M+HCOO]-	821.4693	821.4687	-0.7304	5.97E+08	8.37E+06	
MGDG(18:3;O/3:1)	[M+HCOO]-	629.3179	629.3177	-0.3178	6.90E+07		
MGDG(18:3;O2/16:4)	[M+HCOO]-	821.4693	821.4698	0.6087	1.04E+08	7.89E+07	
MGDG(20:4/5:1;O2) I	[M-H]-	653.3542	653.3534	-1.2245	5.13E+06		1.66E+07
MGDG(20:5/4:1) I	[M+HCOO]-	651.3386	651.3383	-0.4606			1.38E+08
MGDG(20:5/5:1;O)	[M+HCOO]-	681.3492	681.3490	-0.2935			2.60E+07
MGDG(20:5/6:0;O2) I	[M+HCOO]-	713.3754	713.3742	-1.6821			4.36E+07
MGDG(20:5/6:0;O2) II	[M+HCOO]-	713.3754	713.3748	-0.8411			5.60E+07
MGDG(5:2;O/16:0)	[M-H]-	605.3542	605.3550	1.3215			3.95E+07
MGDG(9:1;O2 /16:4)	[M-H]-	653.3542	653.3532	-1.5306	1.47E+08		
Hydroxy fatty acids							
FA 10:0;O	[M-H]-	187.1339	187.1338	-0.7748	2.72E+07	2.12E+07	
FA 10:1;O2 I	[M-H]-	201.1132	201.1132	-0.0497	4.19E+07		
FA 10:1;O2 II	[M-H]-	201.1132	201.1132	-0.0497	8.56E+07	4.75E+07	4.18E+07
FA 10:1;O2 III	[M-H]-	201.1132	201.1132	-0.0497			3.25E+07
FA 13:3;O2 I	[M-H]-	239.1289	239.1287	-0.6691	8.73E+07	1.79E+07	1.26E+06
FA 15:0;O	[M-H]-	257.2122	257.2122	0.0194	1.07E+08	7.18E+07	2.21E+06
FA 18:0;O	[M-H]-	299.2591	299.2589	-0.8187	1.10E+09	9.98E+06	2.64E+06
FA 18:1;O3 I	[M-H]-	329.2333	329.2334	0.3037	9.99E+07	5.51E+07	1.46E+07
FA 18:1;O3 II	[M-H]-	329.2333	329.2334	0.3037	3.90E+07	5.03E+07	1.30E+07
FA 18:2;O I	[M-H]-	295.2278	295.2278	0.0000	7.43E+07	2.81E+08	7.05E+07
FA 18:2;O II	[M-H]-	295.2278	295.2278	0.0000	3.32E+08	5.54E+08	1.23E+08
FA 18:4;O2 I	[M-H]-	307.1915	307.1913	-0.6511	2.63E+07	2.48E+07	
FA11:0;O	[M-H]-	201.1496	201.1496	0.0000	1.44E+08	1.21E+06	
FA16:0;O I	[M-H]-	271.2278	271.2279	0.3687	1.16E+08	3.44E+07	9.98E+07
FA16:1;O I	[M-H]-	269.2122	269.2122	0.0000	4.67E+07	1.33E+07	6.54E+07
FA16:1;O II	[M-H]-	269.2122	269.2122	0.0000	3.76E+07	2.04E+07	1.55E+08
FA16:1;O III	[M-H]-	269.2122	269.2122	0.0000	9.05E+06	1.75E+06	2.21E+07
FA16:1;O IV	[M-H]-	269.2122	269.2122	0.0000	3.12E+07	1.40E+07	8.89E+06
FA16:3;O I	[M-H]-	265.1809	265.1809	0.0189	1.77E+06	2.57E+07	

FA16:3;O II	[M-H]-	265.1809	265.1809	0.0189	1.77E+06	1.77E+07	
FA16:3;O III	[M-H]-	265.1809	265.1809	0.0189	2.96E+07	4.51E+08	3.86E+07
FA16:3;O IV	[M-H]-	265.1809	265.1809	0.0189	2.69E+06	3.24E+07	1.15E+06
FA16:4;O I	[M-H]-	263.1652	263.1652	0.0000	1.98E+07		
FA16:4;O II	[M-H]-	263.1652	263.1652	0.0000	2.10E+07		
FA16:4;O III	[M-H]-	263.1652	263.1652	0.0000	1.70E+08	1.12E+07	
FA16:4;O IV	[M-H]-	263.1652	263.1652	0.0000	1.50E+08	1.32E+07	
FA16:4;O V	[M-H]-	263.1652	263.1652	-0.1710	3.59E+07	1.12E+07	
FA16:4;O VI	[M-H]-	263.1652	263.1652	-0.1710	9.13E+07	3.20E+07	
FA16:4;O VII	[M-H]-	263.1652	263.1652	-0.1710	1.14E+08	4.82E+06	
FA18:2;O3 I	[M-H]-	327.2177	327.2175	-0.5348	5.45E+08	2.34E+07	
FA18:2;O3 II	[M-H]-	327.2177	327.2173	-1.1460	1.77E+08	2.41E+07	
FA18:3;O I	[M-H]-	293.2122	293.2120	-0.6821	1.75E+09	1.32E+09	6.80E+07
FA18:3;O II	[M-H]-	293.2122	293.2120	-0.6821	3.53E+07	5.27E+07	1.62E+06
FA18:3;O III	[M-H]-	293.2122	293.2120	-0.6821	6.79E+07	5.24E+07	1.49E+07
FA18:4;0 I	[M-H]-	291.1965	291.1966	0.3434	2.83E+07		
FA18:4;0 II	[M-H]-	291.1965	291.1966	0.3434	1.00E+08	3.69E+06	1.92E+06
(-)-hydroxycitric acid (FA6:2;O6)	[M-H]-	207.0146	207.0165	9.1781		8.55E+07	
Monoacylglycerophosphates							
LPA(4:0)	[M+HCOO]-	287.0537	287.0537	0.0000	7.43E+07		
Monoacylglycerophosphoethanolamines							
LPE(16:0) I	[M-H]-	452.2782	452.2782	0.0000	2.81E+07	4.43E+07	1.28E+06
LPE(16:0) II	[M-H]-	452.2782	452.2781	-0.2211	1.14E+08	1.68E+08	7.38E+06
LPE(16:1) I	[M-H]-	450.2626	450.2625	-0.2221	2.64E+08	3.65E+08	6.45E+06
LPE(16:1) II	[M-H]-	450.2626	450.2624	-0.4442	7.62E+08	3.25E+08	3.46E+07
LPE(16:3) I	[M-H]-	446.2313	446.2331	4.0338	6.55E+06	2.18E+08	
LPE(16:3) II	[M-H]-	446.2313	446.2312	-0.2241	3.54E+07	1.12E+08	
LPE(17:1)	[M-H]-	464.2782	464.2780	-0.4308	2.03E+07	9.11E+07	
LPE(18:1) I	[M-H]-	478.2939	478.2938	-0.1924	9.37E+06	1.58E+08	2.81E+06
LPE(18:1) II	[M-H]-	478.2939	478.2938	-0.1924	4.36E+07	1.42E+08	4.27E+06
LPE(18:2) I	[M-H]-	476.2782	476.2779	-0.7181	1.94E+07	4.69E+08	
LPE(18:2) II	[M-H]-	476.2782	476.2780	-0.5081	8.66E+07	2.97E+08	2.49E+06
LPE(18:3) I	[M-H]-	474.2626	474.2624	-0.4048	4.55E+07	3.80E+08	
LPE(18:3) II	[M-H]-	474.2626	474.2625	-0.1940	1.43E+08	2.60E+08	
Saturated fatty acids							
C14:0	[M-H]-	227.2016	227.2018	0.8803	3.05E+06		6.38E+07
C15:0	[M+HCOO]-	287.2228	287.2228	0.0000	1.24E+08		
C16:0	[M-H]-	255.2329	255.2328	-0.3918	7.86E+08	9.91E+08	1.71E+09
C17:0	[M-H]-	269.2486	269.2487	0.3714	5.30E+07	2.09E+08	9.44E+06
C18:0	[M-H]-	283.2642	283.2644	0.7061	1.48E+08	2.60E+08	7.23E+07
Unsaturated fatty acids							
C13:3	[M-H]-	207.1390	207.1389	-0.4828	2.60E+08	1.52E+08	1.54E+07
C15:4	[M-H]-	233.1547	233.1545	-0.7720	1.76E+08	1.58E+08	1.26E+08
C16:1	[M-H]-	253.2173	253.2172	-0.3949	2.10E+08	2.94E+08	1.75E+09
C16:2	[M-H]-	251.2016	251.2014	-0.7962	3.07E+07	1.21E+08	3.88E+07

C16:3 I	[M-H]-	249.1860	249.1860	0.0000	3.14E+07	2.55E+08	3.02E+07
C16:4	[M-H]-	247.1703	247.1702	-0.4046	2.25E+08		
C17:1	[M-H]-	267.2329	267.2328	-0.4865	1.28E+07	7.76E+07	2.89E+07
C18:1 I	[M-H]-	281.2486	281.2483	-1.0667	1.44E+09	1.10E+09	7.86E+08
C18:2 I	[M-H]-	279.2329	279.2330	0.3581	4.07E+08	1.39E+09	1.61E+08
C18:3	[M-H]-	277.2173	277.2172	-0.3607	1.22E+09	2.07E+09	9.28E+07
C18:4	[M-H]-	275.2016	275.2017	0.3634	1.43E+08	2.69E+07	3.58E+07
C20:3	[M-H]-	305.2486	305.2483	-0.9828	1.32E+07	3.76E+07	3.75E+07
C20:4	[M-H]-	303.2329	303.2329	0.0000	8.16E+07	3.37E+08	3.84E+08
C20:5	[M-H]-	301.2173	301.2170	-0.9960	9.25E+07	1.44E+08	8.35E+08
C22:5	[M-H]-	329.2486	329.2486	0.0000	9.92E+07	1.24E+08	1.74E+06
C22:6	[M-H]-	327.2329	327.2329	0.0000	1.87E+07	6.33E+07	6.10E+06
			Nucleosides, nucleotides, and analogues				
5′-deoxyribonucleosides							
5'-methylthioadenosine	[M+HCOO]-	342.0878	342.0877	-0.2923	5.67E+07	4.52E+07	2.08E+06
Purine nucleosides							
Adenine	[M-H]-	134.0472	134.0474	1.4920	2.76E+07	6.00E+07	
Adenosine I	[M+HCOO]-	312.0950	312.0948	-0.6408	4.19E+06	5.01E+07	
Inosine I	[M-H]-	267.0735	267.0733	-0.7489	8.54E+07	8.85E+07	6.56E+07
Inosine II	[M-H]-	267.0735	267.0734	-0.3744	5.00E+07	7.48E+07	9.00E+07
Adenosine 2',3'-cyclic phosphate	[M-H]-	328.0452	328.0453	0.3048	1.92E+07	2.04E+08	
Adenosine 5'-monophosphate I	[M-H]-	346.0558	346.0557	-0.2890	8.00E+07	2.70E+07	1.12E+08
Adenosine 5'-monophosphate II	[M-H]-	346.0558	346.0557	-0.2890	4.68E+08	1.71E+09	4.09E+06
Adenosine diphosphate ribose	[M-H]-	558.0644	558.0653	1.6586	4.21E+07	2.59E+08	
Guanosine 3',5'-cyclic monophosphate (cGMP) I	[M-H]-	344.0401	344.0400	-0.2907	1.03E+08	7.02E+07	
Guanosine 3',5'-cyclic monophosphate (cGMP) II	[M-H]-	344.0401	344.0400	-0.2907	1.79E+07	6.01E+08	
Guanosine-5'-monophosphate	[M-H]-	362.0507	362.0505	-0.5508	2.59E+06	1.47E+08	1.83E+06
Pyrimidine nucleosides							
Uridine I	[M-H]-	243.0622	243.0622	0.0000	1.78E+08	1.94E+08	
Thymidine-5'-phosphate I	[M-H]-	321.0493	321.0493	-0.0076	6.44E+06	5.60E+07	2.02E+06
Thymidine-5'-phosphate II	[M-H]-	321.0493	321.0492	-0.3191		9.46E+07	
Uridine 2',3'-cyclic monophosphate; Cyclic 3',5'-uridine monophosphate I	[M-H]-	305.0180	305.0181	0.3278	1.06E+08	5.68E+07	
Uridine 2',3'-cyclic monophosphate; Cyclic 3',5'-uridine monophosphate II	[M-H]-	305.0180	305.0181	0.3203	2.42E+07	5.46E+08	
Uridine 3'-monophosphate	[M-H]-	323.0286	323.0285	-0.3096	1.16E+07	4.74E+08	
Uridine 5'-monophosphate	[M-H]-	323.0286	323.0285	-0.3096	5.64E+08	2.57E+08	5.31E+07

Ribonucleoside 3′-phosphates							
Adenosine 3'-monophosphate (3'-AMP) I	[M-H]-	346.0558	346.0557	-0.2890	5.00E+06	3.90E+08	
Guanosine-3'-monophosphate I	[M-H]-	362.0507	362.0506	-0.2746		2.42E+08	
Guanosine-3'-monophosphate II	[M-H]-	362.0507	362.0508	0.2778		1.58E+08	
Amino acids, peptides, and analogues							
Asparagine	[M-H]-	131.0462	131.0461	-0.7631	1.79E+08	1.94E+08	
Aspartic acid	[M-H]-	132.0302	132.0303	0.7574	1.46E+08	1.12E+08	2.21E+07
Citrulline	[M-H]-	174.0884	174.0885	0.5744	6.04E+07	3.85E+07	
Cysteic acid	[M-H]-	167.9972	167.9967	-2.9762	6.99E+07	3.36E+07	
Diaminopimelic acid	[M-H]-	189.0881	189.0881	0.0000	1.72E+06	1.03E+07	
Glutathione (oxidized)	[M-H]-	611.1447	611.1446	-0.1636	4.99E+07		
Glutathionesulfonic acid I	[M-H]-	354.0612	354.0612	0.0000	8.54E+08	1.83E+07	
Homocysteic acid	[M-H]-	182.0128	182.0135	3.8459	1.29E+08	9.93E+07	
L-Glutamic acid	[M-H]-	146.0459	146.0458	-0.6847	1.21E+09	8.78E+08	2.98E+08
N-Undecanoylglycine	[M-H]-	242.1761	242.1761	0.0000	7.80E+07	8.20E+07	4.98E+07
Serine	[M-H]-	104.0353	104.0352	-0.9612	3.02E+07	4.40E+07	
Threonine;allo-threonine	[M-H]-	118.0509	118.0509	0.0000	4.91E+07	9.44E+07	1.21E+06
Organic acids and derivatives							
Citric acid I	[M-H]-	191.0197	191.0199	1.0470		7.43E+06	1.04E+09
Citric acid II	[M-H]-	191.0197	191.0197	0.0000		2.97E+08	6.62E+08
Glycerophosphoglycerol	[M-H]-	245.0432	245.0431	-0.4081	8.88E+07	3.53E+09	8.05E+06
L-lactic acid; 3-hydroxypropionic acid	[M-H]-				2.56E+07	3.20E+07	
Malic acid	[M-H]-	133.0142	133.0142	0.0000	4.37E+08	9.12E+08	7.51E+06
Succinic acid; methylmalonic acid III	[M-H]-	117.0193	117.0193	0.0000	1.21E+09	1.81E+09	5.65E+08
Carbohydrates and carbohydrate conjugates							
Arabinonic acid; *D*-Lyxonic acid; *D*-Ribulose; Ribonic acid; *D*-xylonic acid	[M-H]-	165.0404	165.0404	0.0000	2.95E+08	5.23E+08	4.20E+07
Cytidine-3'-monophosphate	[M-H]-	322.0446	322.0447	0.3105	1.94E+06	2.84E+07	
D-Glucosamine 6-phosphate	[M-H]-	258.0384	258.0385	0.3875	1.31E+07	1.48E+08	
Disaccharide	[M+HCOO]-	387.1144	387.1142	-0.5166	2.76E+09	1.51E+09	
D-Threonic acid	[M-H]-	135.0299	135.0315	11.8492		2.60E+08	
Gluconic acid I	[M-H]-	195.0510	195.0510	0.0000	1.32E+08	7.55E+07	2.18E+07
Gluconic acid II	[M-H]-	195.0510	195.0510	0.0000	3.27E+07	2.46E+07	
L-Threonic acid	[M-H]-	135.0299	135.0299	0.0000	7.02E+08	7.55E+08	6.23E+08

Roman numbering represents different isomers; FA, fatty acid, DGDG, digalactosyldiacylglycerol; DGMG, digalactosylmonoacylglycerol; DGTS, diacylglyceryl-*O*-4′-(N,N,N-trimethyl) homoserine; LPA, monoacylglycerophosphate; LPC, lysophosphatidylcholine, LPE, lsyophosphatidylethanolamine; MGMG, Monogalactosyl monoacylglycerol; MGDG, Monogalactosyldiacylglycerol; MGTS, monoacylglyceryl-*O*-4′-(N,N,N-trimethyl) homoserine; PE, phosphatidylethanolamine.

References

1. Kessner, D.; Chambers, M.; Burke, R.; Agus, D.; Mallick, P. ProteoWizard: open source software for rapid proteomics tools development. *Bioinformatics* **2008**, *24*, 2534-2536.
2. Pluskal, T.; Castillo, S.; Villar-Briones, A.; Orešič, M. MZmine 2: Modular framework for processing, visualizing, and analyzing mass spectrometry-based molecular profile data. *BMC Bioinformatics* **2010**, *11*, 395.
3. Myers, O.D.; Sumner, S.J.; Li, S.; Barnes, S.; Du, X. One step forward for reducing false positive and false negative compound identifications from mass spectrometry metabolomics data: new algorithms for constructing extracted ion chromatograms and detecting chromatographic peaks. *Anal Chem* **2017**, *89*, 8696-8703.
